# Ophthalmoscopic Examination of the Eyes of the Hypnotized

**Published:** 1890-11

**Authors:** J. Falk


### OPHTHALMOSCOPIC EXAMINATION OF THE EYES OF THE HYPNOTIZED.

DRS. LUYS AND BACCHI, Members of the Paris Academy of Sciences, (*Centralblatt für Praktische Augenheilkunde,*) say they have employed the ophthalmoscope for the examination of the fundus of the eyes of hypnotized patients. Six women and three men were examined in the stadium of catalepsia, somnambulismos, and in the condition of Foxination.

In catalepsy they observed a change in the pale color of the retina, the papillæ assumed a rose-red color, and the vessels were much enlarged. During the whole period of the cataleptic condition the hyperemia of the papillæ continued, the pupils were largely dilated and contracted slightly when exposed to bright light.

In Foxination the peculiar condition of hyperemia continued, as in catalepsy.

In the stadium of somnambulism there was little change in the appearance of the blood-vessels at the fundus ; the papillæ being less intensely colored, and the pupils more mobile and sympathetic to light.

In catalepsy the eyes are immobile, whereas in somnambulism the eyes soon regain their mobility.

The ophthalmoscopic examination of a patient in the hypnotic condition has also a forensic interest, because the condition cannot be simulated, and provides a means to diagnosticate the hypnotic condition.

The authors conclude their investigation with the remark that in both conditions the physiological functions of the eye are increased and the acuity of vision more than normal. (?)

J. Falk.

